# Involvement of the Choroid Plexus in the Pathogenesis of Niemann-Pick Disease Type C

**DOI:** 10.3389/fncel.2021.757482

**Published:** 2021-10-15

**Authors:** Lien Van Hoecke, Caroline Van Cauwenberghe, Kristina Dominko, Griet Van Imschoot, Elien Van Wonterghem, Jonas Castelein, Junhua Xie, Wouter Claeys, Charysse Vandendriessche, Anna Kremer, Peter Borghgraef, Riet De Rycke, Silva Hecimovic, Roosmarijn E. Vandenbroucke

**Affiliations:** ^1^VIB Center for Inflammation Research, VIB, Ghent, Belgium; ^2^Department of Biomedical Molecular Biology, Ghent University, Ghent, Belgium; ^3^Division of Molecular Medicine, Ruder Boskovic Institute, Zagreb, Croatia; ^4^Hepatology Research Unit, Department of Internal Medicine and Pediatrics, Liver Research Center Ghent, Ghent University, Ghent, Belgium; ^5^VIB BioImaging Core Ghent, VIB, Ghent, Belgium; ^6^Ghent University Expertise Centre for Transmission Electron Microscopy, Ghent, Belgium

**Keywords:** Niemann-Pick type C disease, choroid plexus, extracellular vesicles, blood-CSF-barrier, autophagosomes

## Abstract

Niemann-Pick type C (NPC) disease, sometimes called childhood Alzheimer’s, is a rare neurovisceral lipid storage disease with progressive neurodegeneration leading to premature death. The disease is caused by loss-of-function mutations in the *Npc1* or *Npc2* gene which both result into lipid accumulation in the late endosomes and lysosomes. Since the disease presents with a broad heterogenous clinical spectrum, the involved disease mechanisms are still incompletely understood and this hampers finding an effective treatment. As NPC patients, who carry NPC1 mutations, have shown to share several pathological features with Alzheimer’s disease (AD) and we and others have previously shown that AD is associated with a dysfunctionality of the blood-cerebrospinal fluid (CSF) barrier located at choroid plexus, we investigated the functionality of this latter barrier in NPC1 pathology. Using NPC1^–/–^ mice, we show that despite an increase in inflammatory gene expression in choroid plexus epithelial (CPE) cells, the blood-CSF barrier integrity is not dramatically affected. Interestingly, we did observe a massive increase in autophagosomes in CPE cells and enlarged extracellular vesicles (EVs) in CSF upon NPC1 pathology. Additionally, we revealed that these EVs exert toxic effects on brain tissue, *in vitro* as well as *in vivo*. Moreover, we observed that EVs derived from the supernatant of NPC1^–/–^ choroid plexus explants are able to induce typical brain pathology characteristics of NPC1^–/–^, more specifically microgliosis and astrogliosis. Taken together, our data reveal for the first time that the choroid plexus and CSF EVs might play a role in the brain-related pathogenesis of NPC1.

## Introduction

Niemann-Pick disease type C (NPC) is a highly heterogeneous rare lipid storage disorder with an incidence estimated at about 1 in 100,000 individuals (Vanier, 2010). The disease is characterized by the accumulation of unesterified cholesterol and other lipids in the late endosomes and lysosomes (LE/Lys) and is caused by loss-of-function mutation in the *Npc1* (95%) or *Npc2* (5%) gene. The corresponding proteins yield deficient function in the binding and transport of intracellular cholesterol, resulting in cholesterol accumulation within LE/Lys. As every cell is affected by an impaired cholesterol trafficking mechanism, NPC pathology affects basically every organ. Yet, some organs show worse clinical features than others. Especially the liver, spleen and lungs but also the brain are heavily affected.

The clinical presentation of NPC1 is quite heterogenous and the pathology manifests itself mainly during childhood, but also juvenile and adult cases are reported (Shulman et al., 1995; Garver et al., 2007; Patterson et al., 2017; Bonnot et al., 2019a, b). While visceral manifestations tend to predominate during the perinatal and infantile period, neurological and psychiatric involvement is more prominent during the juvenile/adult period (Mengel et al., 2013). Following a long term gradual neurological decline, the death of the NPC1 patient is often the fatal result (Mengel et al., 2013). In order to find an effective treatment to slow down or halt the disease, better understanding of the mechanisms responsible for neurological decline are needed.

So far it is shown that loss of NPC1 functionality seriously affects different components of the central nervous system (CNS). E.g., in neurons, there is storage of glycolipids and free sphingosine resulting in the formation of neurofibrillary tangles as well as neuroaxonal dystrophy and subsequent neuronal death (Vanier, 1999). Moreover, losses in Purkinje cells of the cerebellum, hypomyelination, axonal spheroids and abundancy of cholesterol accumulated astrocytes in selective brain regions are linked to NPC1 pathology. Furthermore, alterations in the concentration of neurotransmitters have been detected in specific brain regions which can cause seizures (Karten et al., 2009; McCauliff et al., 2015). Recently Colombo et al. (2021) showed that loss of NPC1 enhances phagocytic uptake and impairs lipid trafficking in microglia. Despite all these findings, the ultimate link between NPC1 and neurodegeneration is not fully understood yet.

NPC1 disease has shown to share several pathological features with a more common neurodegenerative disorder, namely Alzheimer’s disease (AD) (Malnar et al., 2014). For this reason, NPC1 disease is sometimes called childhood Alzheimer’s. We have previously shown that AD is associated with a dysfunctionality of the blood-cerebrospinal fluid (CSF) barrier (Vandenbroucke et al., 2012; Brkic et al., 2015; Steeland et al., 2018). This latter barrier is located at the choroid plexus, a highly vascularized structure protruding in the ventricles of the brain. The blood-CSF barrier consists of a monolayer of choroid plexus epithelial (CPE) cells that are firmly interconnected by tight junctions and are situated at the interface between blood and the CSF-containing ventricular cavities (Vandenbroucke, 2016). It assures a balanced and well-controlled micro-environment in the CNS, providing protection against external insults such as toxins, infectious agents and peripheral blood fluctuations. In the present study, we studied the functionality of the blood-CSF barrier in NPC1 pathology.

To investigate this, we used a mouse model that carries a spontaneous loss of function mutation within the *Npc1* gene (deletion of 11 out of its 13 transmembrane domains, BALB/cNctr-Npc1m1N/J) (Pentchev et al., 1980; Loftus et al., 1997; Colombo et al., 2021). This model represents features of early onset human pathology, including neurodegeneration of vulnerable NPC regions, such as Purkinje cells in the cerebellum or other neurons in the thalamus (Tanaka et al., 1988; Higashi et al., 1993). We observed that NPC1^–/–^ brain pathology is associated with an increased inflammatory gene expression in choroid plexus tissue. Surprisingly, this inflammatory status only has limited effect on the CPE cell morphology and tight junction protein levels and this was associated with maintenance of blood-CSF barrier integrity. This result is in shear contrast to our previous results in an Alzheimer’s disease setting where we linked an increased inflammatory status of CPE cells to a loss of blood CSF barrier integrity (Vandenbroucke et al., 2012; Brkic et al., 2015; Gorle et al., 2016). Interestingly, we observed a significant increase in autophagosomes in CPE cells and larger extracellular vesicles (EVs) in the CSF of NPC1^–/–^ mice. Moreover, we could link these differences in EVs with typical NPC1 brain pathology phenotypes. More precisely, injection of EVs obtained from choroid plexus explants of NPC1^–/–^ mice in the brain of wild type mice is sufficient to induce microgliosis and astrogliosis, typical CNS features of NPC1 pathology.

## Results

### NPC1^–/–^ Mice Display Limited Inflammation in the Choroid Plexus

**FIGURE 1:**
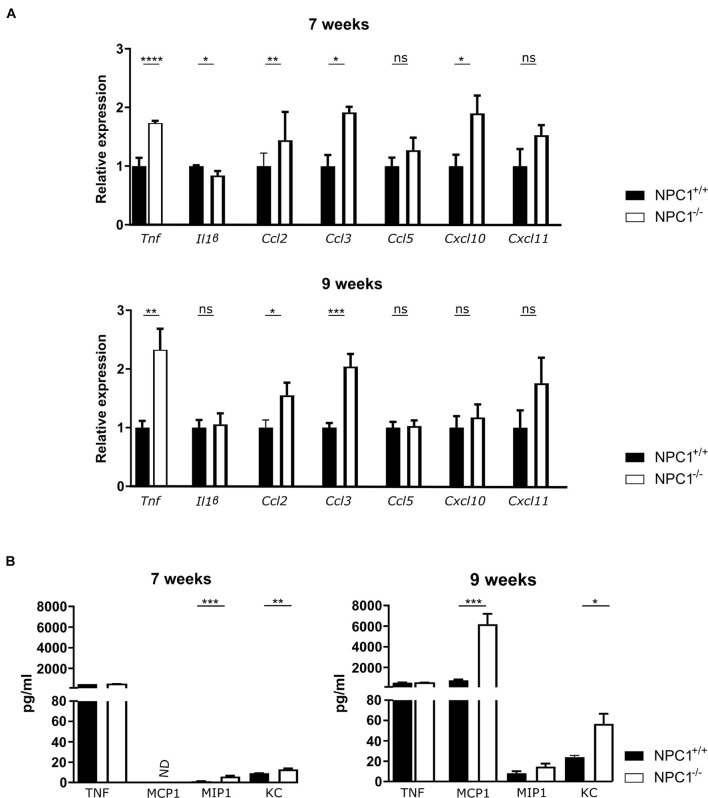
Increased cytokine and chemokine levels in the choroid plexus and CSF of NPC1^–/–^ mice. **(A)** qRT-PCR analysis of choroid plexus isolated from NPC1^+/+^ (*n* = 11; black) and NPC1^–/–^ (*n* = 13; white) of 7 and 9 weeks old mice. Results are represented relative to the NPC1^+/+^ condition. **(B)** Bioplex analysis of protein levels in CSF from NPC1^+/+^ (black) and NPC1^–/–^ (white) mice (*n* = 5). Data are shown as mean ± SEM. Statistical analyses on datasets were performed by Mann–Whitney test. Asterisks indicate statistical significance (*p < 0.05, **p < 0.01, ***p < 0.001, ****p < 0.0001, ns: non-significant). ND: not detected.

Central nervous system inflammation is a common feature of neurodegenerative diseases, including lysosomal storage disorders (Perry et al., 2007). Increasing evidence, including our own research, supports an important role for the choroid plexus in inflammatory diseases such as sepsis and AD (Vandenbroucke et al., 2012; Brkic et al., 2015; Demeestere et al., 2015; Balusu et al., 2016a, b; Vandenbroucke, 2016). Moreover, the choroid plexus is believed to be an entry gate of leukocytes into the brain (Demeestere et al., 2015). In this regard we looked into the inflammatory state of the choroid plexus of NPC1 diseased mice at intermediate and end-stage of the disease (at 7 and 9 weeks of age, respectively) (Figure 1A). Gene expression analysis revealed that several inflammatory genes were significantly upregulated in the choroid plexus of NPC1^–/–^ mice. The expression levels of *Ccl2* and *Ccl3*, both important for the recruitment and activation of leukocytes, are significantly increased in 7 and 9 weeks old NPC1^–/–^ mice compared to wild type littermates. Also, the expression levels of other chemokines (*Ccl5*, *Cxcl10*, and *Cxcl11*) were increased but this increase was not significant. *Tnf*, a major inflammatory cytokine, showed increased gene expression in the choroid plexus of NPC1^–/–^ mice at both 7 and 9 weeks.

Next to an increase in the typical pro-inflammatory cytokines, also an increase in expression of interferon stimulation response element (ISRE) genes can be observed in the choroid plexus of NPC1 deficient mice (Supplementary Figure 1).

The increase that is seen in gene expression of inflammatory cytokines and chemokines is less well pronounced on protein level (Figure 1B). This can be explained by the fact that the protein levels are too diluted in the CSF.

Taken together, our data show that NPC1^–/–^ pathology is associated with limited inflammation in the choroid plexus.

### NPC1^–/–^ Pathology Has No Effect on Blood-Cerebrospinal Fluid Barrier Integrity

NPC1 disease has shown to share several pathological features with AD, a common neurodegenerative disorder (Malnar et al., 2014). We have previously shown that AD is associated with a limited increased inflammation in choroid plexus, comparable to what we observe here in NPC1^–/–^ mice (Figure 1; Vandenbroucke et al., 2012; Brkic et al., 2015; Steeland et al., 2018). Moreover, we demonstrated that in AD pathology the increase in inflammation affects the blood-CSF barrier integrity. Therefore, we next analyzed the blood-CSF barrier integrity in NPC1^–/–^ mice using qPCR and immunostaining. We determined whether changes in junctional proteins in the choroid plexus can be observed in 7 or 9 weeks old NPC1^–/–^ mice compared to wild type littermates focusing on Zonula occludens-1 (ZO1), Claudin5 (CLDN5), Occludin (OCLN), and E-cadherin (CDH1).

**FIGURE 2:**
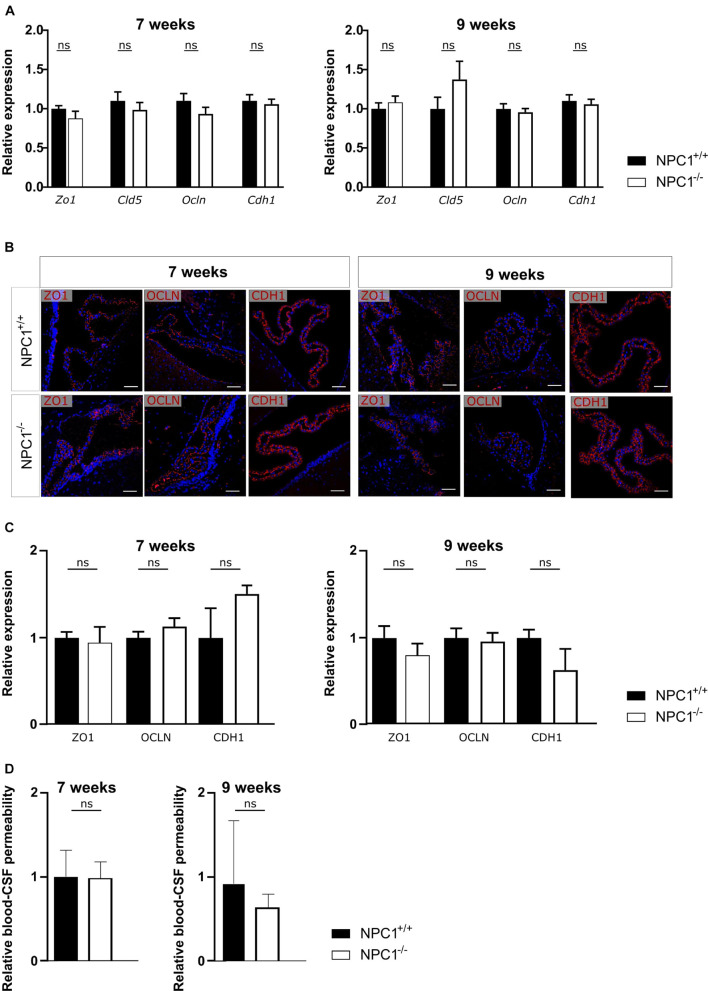
NPC1^–/–^ pathology has no effect on blood-cerebrospinal fluid (CSF) barrier integrity. **(A)** qRT-PCR analysis of choroid plexus isolated from NPC1^+/+^ and NPC1^–/–^ of 7 and 9 weeks old mice (*n* = 11–13). **(B)** Representative images of ZO1, OCLN, and CDH1 staining (red) of the choroid plexus of NPC1^+/+^ and NPC1^–/–^ mice of 7 and 9 weeks old. Cell nuclei are counterstained with Hoechst (blue) (*n* = 4–5). Scale bar represents 20 μm. **(C)** Quantification of the ZO1, OCLN, or CDH1 signal at the tight junctions of the choroid plexus epithelial cells. **(D)** Relative blood-CSF barrier permeability of 7 and 9 weeks old NPC1^+/+^ and NPC1^–/–^ mice of (*n* = 5–7). Data are shown as mean ± SEM. Statistical analyses on datasets were performed by Mann–Whitney test (ns: non-significant).

Gene expression analysis revealed that none of the examined tight junctions is affected by the NPC1 pathology (Figure 2A). Moreover, at both ages also the tight junction protein levels and their subcellular localization are comparable between NPC1^–/–^ and NPC1^+/+^ mice (Figures 2B,C). In line with these results, we show that there is no difference in leakiness of the blood-CSF barrier between NPC1^–/–^ and NPC1^+/+^ mice (Figure 2D). For the latter, we injected 4 kDa FITC-dextran (4FD) intravenously, followed by CSF isolation 15 min later. Next, determining the fluorescent signal of 4FD in the CSF is a measure for blood-CSF barrier integrity as in healthy conditions 4FD is unable to cross the blood-CSF barrier.

Collectively and in contrast to what was expected, NPC1 pathology is not associated with a loss in blood-CSF barrier integrity despite the limited increase in inflammatory gene expression.

### NPC1^–/–^ Pathology Is Associated With an Increase in Autophagosomes in the Choroid Plexus Epithelial Cells

**FIGURE 3:**
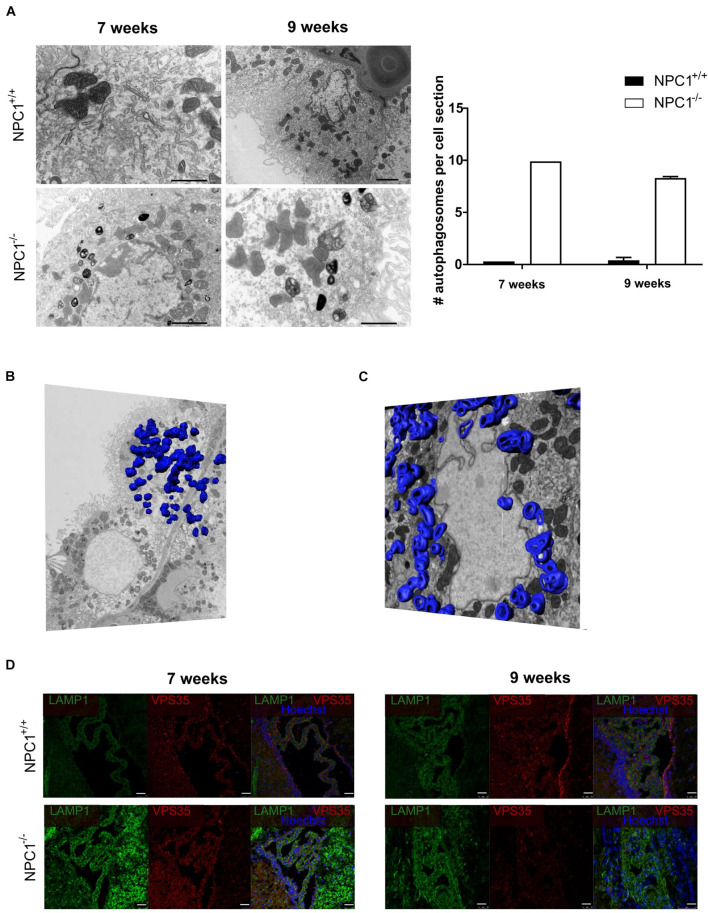
NPC1^–/–^ mice show a huge increase in autophagosomes in the choroid plexus epithelial cells. **(A)** Representative transmission electron microscopy (TEM) images of choroid plexus epithelial cells of the fourth ventricle of NPC1^+/+^ and NPC1^–/–^ mice (7 and 9 weeks old). Scale bar represents 10 μm. The graph represents the amount of autophagosomes per cell section in 7 and 9 weeks old NPC1^+/+^ (black) and NPC1^–/–^ (white) mice (*n* = 3). **(B)** Representative serial block face scanning electron microscope (SBF-SEM) snapshot of Supplementary Video 1 of a choroid plexus epithelial cell of the fourth ventricle of a 9 weeks old NPC1^–/–^ mouse. The autophagosomes are traced on the different sections and visualized in blue (*n* = 1). **(C)** Representative focused ion beam scanning electron microscopy (FIB-SEM) snapshot of Supplementary Video 2 of a choroid plexus epithelial cell of the fourth ventricle of a 9 weeks old NPC1^–/–^ mouse. The autophagosomes are traced on the different sections and visualized in blue (*n* = 1). **(D)** Representative images of LAMP1 staining (green) and VPS35 (red) of the choroid plexus of the fourth ventricle of NPC1^+/+^ and NPC1^–/–^ mice of 7 and 9 weeks old. Cell nuclei are counterstained with Hoechst (blue) (*n* = 4–5). Scale bar represents 25 μm.

Previous research showed that the choroid plexus undergoes morphological alterations in AD (Marques et al., 2013; Brkic et al., 2015). Therefore we studied CPE cell morphology of NPC1^–/–^ mice compared to NPC1^+/+^ mice by using transmission electron microscopy (TEM). These images revealed a remarkable increase in number of autophagosomes per CPE cell in NPC1^–/–^ mice compared to wild type littermates (Figure 3A). This significant increase is seen both at 7 and 9 weeks of age. Autophagy is a kind of waste elimination system to maintain cellular homeostasis through clearance and recycling of damaged proteins and organelles from the cytoplasm to autophagosomes and then to lysosomes (Klionsky and Emr, 2000; Takahashi et al., 2017).

To have a closer look at the autophagosomes in the CPE cells we performed serial block face scanning electron microscopy (SBF-SEM) (Figure 3B and Supplementary Video 1) and focused ion beam scanning electron microscopy (FIB-SEM) (Figure 3C and Supplementary Video 2). Both technologies use a fully automated microtome installed in the SEM chamber to produce serial sections which allows to make 3D reconstructions of the analyzed sample (Denk and Horstmann, 2004). This revealed the presence of an incredible amount of autophagosomes in CPE cells of NPC1^–/–^ mice. To visualize these autophagosomes in the SBF-SEM and FIB-SEM images in 3D, we manually traced the autophagosomes using the MIB software for ∼500 sections per sample followed by merging to generate a 3D model of the autophagosomes in the CPE cells.

Next, we confirmed the higher amount of autophagosomes in the CPE cells of NPC1^–/–^ mice via immunofluorescent stainings against the autophagosome markers lysosomal-associated membrane protein 1 (LAMP1) and vacuolar protein sorting-associated protein 35 (VPS35) (Figure 3D). At 7 and 9 weeks a clear increase in expression of LAMP1, a protein crucial for the maintenance of lysosomal integrity, can be seen in NPC1^–/–^ mice compared to wild type littermates. Also, the expression of VPS35, a protein involved in autophagy, is increased in the choroid plexus of NPC1^–/–^ mice of 7 weeks. Taken together, a significant increase in autophagosomes in the CPE cells of NPC1^–/–^ mice is observed compared to CPE cells of wild type littermates.

### NPC1^–/–^ Pathology Is Associated With Larger and Less Extracellular Vesicles in the Cerebrospinal Fluid

Recently, we revealed a novel way of blood-brain communication activated by inflammation namely by secretion of CPE-derived EVs into the CSF (Balusu et al., 2016b). Moreover, evidence was provided that the CPE cells are an important source of the CSF EVs. As we observed changes in the autophagosome pathway (Figure 3) which is intertwined with the endosomal/exosomal pathway (Salimi et al., 2020), we next investigated EV changes in the CSF in NPC1^–/–^ mice. Similar to autophagosomes that transport damaged materials to lysosomes for degradation, vesicles generated from endocytosis and endosomal compartment transport damaged molecules to lysosomes or expel them out of the cell via exosomes (Salimi et al., 2020).

**FIGURE 4:**
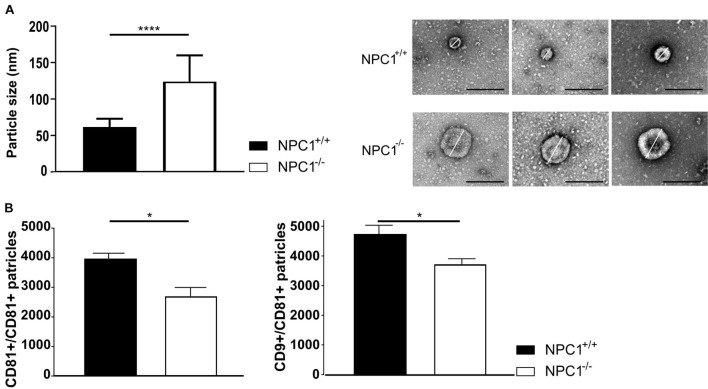
NPC1^–/–^ mice contain larger and less CSF extracellular vesicles (EVs). **(A)** Average size and representative negative staining transmission electron microscopy (TEM) images of CSF EVs from 9 weeks old NPC1^+/+^ (black; *n* = 30) and NPC1^–/–^ (white: *n* = 30) mice. Scale bar represents 500 nm. The size represents the mean ± SEM of 30 EVs per condition. **(B)** ExoView analysis of the amount of CD81 captured and CD9 captured–CD81 positive EVs in CSF of NPC1^+/+^ and NPC1^–/–^ mice of 9 weeks old mice. Bar represent mean ± SEM of four biological replicates. For each biological replicate, the presented result is the average from three technical replicates on the chip. Data are shown as a mean ± SEM. Statistical analyses on datasets were performed by Mann–Whitney test. Asterisks indicate statistical significance (*p < 0.05 and ****p < 0.0001, ns: non-significant).

We performed a negative staining TEM on the CSF and subsequent imaging demonstrates a significant increase in particle size from ±60 nm in CSF of NPC1^+/+^ mice to ±123 nm in CSF of NPC1^–/–^ mice (Figure 4A). Following, we performed ExoView analysis on the CSF samples to quantify the number of EVs (Figure 4B). The ExoView technology captures specifically EVs based on the expression of tetraspanins (CD81 and CD9). This technique revealed a decrease of the amount of CD9 and CD81 positive EVs in CSF of NPC1^–/–^ mice compared to wild type litter mates.

Our data show that larger but less EVs are present in the CSF of NPC1^–/–^ mice compared to the CSF of wild type littermates. Thus, in NPC1 pathology the observed enhanced inflammatory status of choroid plexus (Figure 1) is not linked with increased secretion of EVs in the CSF (Figure 4).

### Extracellular Vesicles in Cerebrospinal Fluid of NPC1^–/–^ Mice Have Pathologic Effects on the Brain

**FIGURE 5:**
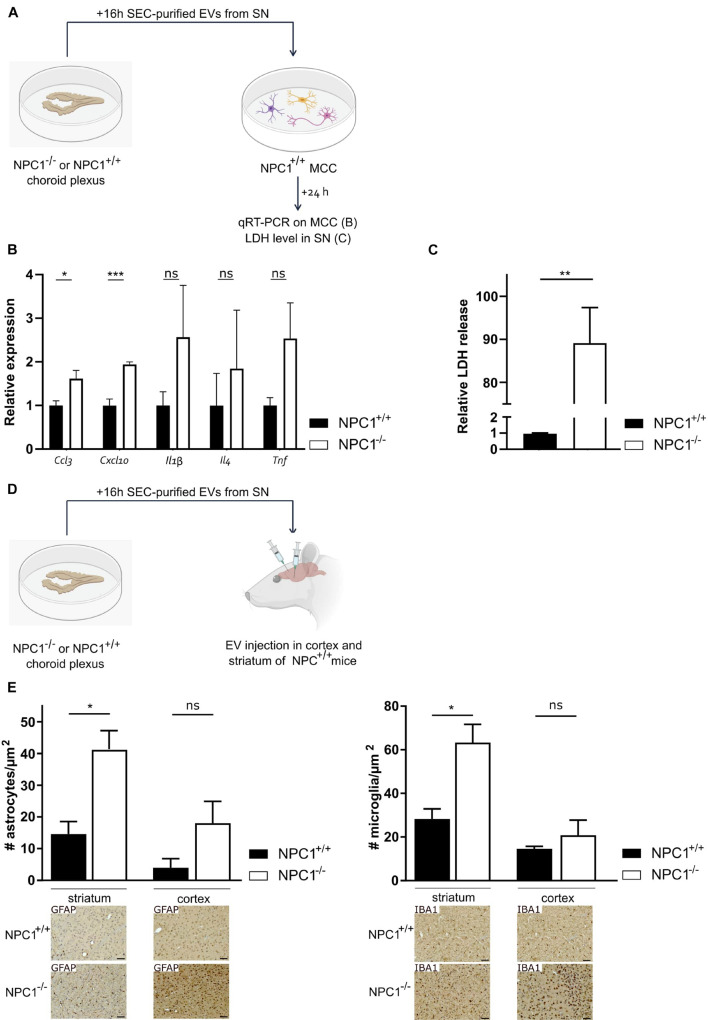
Extracellular vesicles (EVs) isolated from choroid plexus explants of NPC1^–/–^ mice have toxic effects on the brain. **(A)** Overview of the experimental setup. Choroid plexus explants were isolated from of 9 weeks old NPC1^+/+^ and NPC1^–/–^ mice (*n* = 5) and cultured for 16 h in Opti-MEM, after which the supernatant was collected followed by EV purification. MCCs were incubated with size exclusion chromatography (SEC) purified EVs separated from the secretome of the choroid plexus explants. 24 h after incubation qRT-PCR was performed on the MCC **(B)** and supernatant was collected and analyzed for LDH levels **(C)**. **(D)** Overview of the experimental setup. choroid plexus explants were isolated from 9 weeks old NPC1^+/+^ and NPC1^–/–^ mice (*n* = 3) and cultured for 16 h in Opti-MEM, after which the supernatant was collected. SEC-purified EVs separated from the secretome of choroid plexus explants were injected in the striatum or cortex of NPC1^+/+^ mice followed by analysis 3 days later. **(E)** Quantification of IBA1+ (microglia) and GFAP+ (astrocytes) cells per μm^2^ in brain sections of wild type mice injected with EVs isolated from NPC1^+/+^ or NPC1^–/–^ choroid plexus explants. Representative images are shown below the graphs. Scale bar represents 20 μm. Data are shown as a mean ± SEM. Statistical analyses on datasets were performed by Mann–Whitney test in panels **(B,C)** and *t*-test in panel **(E)**. Asterisks indicate statistical significance (*p < 0.05, **p < 0.01, ***p < 0.001, ns: non-significant).

Previously, we showed that upon systemic inflammation the choroid plexus transmits information about the peripheral inflammatory status to the CNS via the release of EVs into the CSF (Balusu et al., 2016b). Here, we investigated whether the changes in CSF EVs that we observed (Figure 4) play a role in the brain pathology of NPC1^–/–^ mice. To address this, we prepared choroid plexus explants from NPC1^–/–^ and NPC1^+/+^ mice of 9 weeks and purified EVs from the choroid plexus explant supernatant via size exclusion chromatography (Figure 5A). The quality of the EV fractions were verified as we reported before (Vandendriessche et al., 2021) and according to the MISEV18 guidelines (Thery et al., 2018). Next, purified EVs were transferred to mixed cortical cultures (MCCs) of NPC1^+/+^ mice and 24 h later, both MCC cells and cell supernatant were analyzed. This revealed that *Ccl3* and *Cxcl10* gene expression levels were significantly higher in MCC cells incubated with EVs derived from NPC1^–/–^ choroid plexus explants compared to MCC cells incubated with EVs derived from NPC1^+/+^ choroid plexus explants (Figure 5B). Also, the expression levels of other chemokines, *Il-1*β, *Il4*, and *TNF*, were elevated but this increase was not significant. Additionally, we also analyzed the lactate dehydrogenase (LDH) levels, an enzyme that is secreted upon cell death, in the MCC supernatant after incubation with NPC1^+/+^ versus NPC1^–/–^ EVs. Strikingly, a significant increase in LDH was detected in the supernatant of MCC incubated with EVs derived from NPC1^–/–^ choroid plexus explants (Figure 5C). This further underlines the toxic effect of EVs derived from NPC1^–/–^ mice on brain cells.

Next, we looked into the *in vivo* effect of EVs derived from NPC1^–/–^ choroid plexus explants (Figures 5D,E). Again, choroid plexus explants were prepared from 9 weeks old NPC1^–/–^ and NPC1^+/+^ mice followed by EV purification from the supernatant. Next, the SEC-purified EVs were injected into the striatum and prefrontal cortex (PFC) of NPC1^+/+^ mice. Three days later, we performed immunostainings on the brain of these mice to investigate the effect of the injected EVs on the number of astrocytes and microglia in the striatum and PFC. Astrocytosis and microgliosis, typical features of NPC1 pathology, was clearly observed in the striatum and PFC of wild type mice injected with EVs derived from NPC1^–/–^ choroid plexus explants while this was not observed in mice injected with EVs derived from NPC1^+/+^ choroid plexus explants (Figure 5E). Moreover, we performed TUNEL stainings on these brain sections to visualize dying cells. No clear TUNEL positive signal could be observed (Supplementary Figure 2).

Taken together, our *in vitro* and *in vivo* data show that EVs derived from NPC1^–/–^ choroid plexus explants have pathologic effects on brain cells.

## Discussion

Pathology within the CNS is a feature of most lysosomal storage diseases, probably because neurons are uniquely vulnerable to perturbation of normal lysosomal activity. NPC disease is a rare neurodegenerative disorder caused by mutations in *Npc1* or *Npc2* gene, resulting in abnormal late endosomal and lysosomal (LE/Lys) lipid storage. Despite the fact that different neurological impairments are described (Vanier, 1999, 2010; Karten et al., 2009; McCauliff et al., 2015; Colombo et al., 2021), the involved disease mechanisms are still incompletely understood. This makes finding an effective treatment very difficult.

NPC1 disease causes children to have memory loss and other symptoms commonly associated with AD. For this reason the term childhood Alzheimer’s is often used to refer to NPC1 disease (Malnar et al., 2014). As we have previously shown that AD is associated with a dysfunctionality of the blood-CSF barrier (Vandenbroucke et al., 2012; Brkic et al., 2015; Steeland et al., 2018), we investigated if the same hold true in NPC1 pathology. The blood-CSF barrier is located at the choroid plexus and is one of the brain barriers that assures a balanced and well-controlled micro-environment in the CNS. The choroid plexus’ most important functions next to the formation of a barrier are transport of nutrients, ions, gases and metabolites between the fenestrated choroidal blood vessels and the CNS, the secretion and production of CSF and the detoxification of that CSF. Recently, we and others reported that CPE cells are able to secrete EVs, including exosomes, into the CSF (Grapp et al., 2013; Balusu et al., 2016b; Lepko et al., 2019). Moreover, this EV production is affected in response to systemic inflammation and the EVs can transfer pro-inflammatory messages to recipient brain cells (Balusu et al., 2016b).

Increasing evidence, including our own research, show that in a broad range of diseases, the choroid plexus is inflamed (Vandenbroucke et al., 2012; Brkic et al., 2015; Steeland et al., 2018). This inflammatory status may affect the functionality of the choroid plexus including its barrier integrity and its secretory capacity. Comparable to our results in AD mouse models, we observed that *Npc1* dysfunctionality is associated with increased inflammation in the choroid plexus tissue. Important to mention is that no relevant sex differences could be observed regarding the inflammatory status of the choroid plexus. Consequently, gender-balanced groups were used in all follow up experiments. Next to the typical pro-inflammatory cytokines that we tested, such as TNF, IL-1β, CCL2, CCL3, CCL5, CXCL10, CXCL11 also type I IFNs have shown to play important roles in inflammation, both outside and inside the CNS (Axtell and Steinman, 2008; Prinz et al., 2008; Hofer and Campbell, 2013; Thery et al., 2018). It is known from literature that a type I interferon dependent gene expression profile can be found in the choroid plexus of aged mice (Baruch et al., 2014) and in mouse models of AD (Mesquita et al., 2015). This profile is linked with a decrease in brain plasticity, which is needed for physiological immune-dependent brain maintenance and resolution of neuroinflammation in aging and pathology (Ziv et al., 2006; Kipnis et al., 2012; Baruch et al., 2013; Kunis et al., 2013; Shechter et al., 2013; Schwartz and Baruch, 2014). Here, we show that also in NPC1 deficient mice, an increase in expression of ISRE genes can be observed in the choroid plexus. Strikingly, this increase in expression of typical pro-inflammatory cytokines and ISRE genes is not linked to a loss of blood-CSF barrier integrity, analyzed via gene expression analysis, immunostainings and blood-CSF barrier permeability assay. This result is in shear contrast to previous work in which we observed that increased inflammation at the choroid plexus negatively impacted barrier function. Importantly, the cyto- and chemokine increase that we observed in choroid plexus tissue in the NPC1^–/–^ mice was much lower compared to Alzheimer’s (Brkic et al., 2015) and endotoxemic mice, which might explain this discrepancy.

Next, TEM analysis did reveal a tremendous accumulation of autophagosomes in the CPE cells of NPC1^–/–^ mice. Additionally, we made SBF-SEM and FIB-SEM 3D images to have a closer look at the autophagosomes in the CPE cells and showed an incredible amount of autophagosomes in CPE cells of NPC1^–/–^ mice. It is known that autophagy and LE/Lys secretory pathways are important ways to degrade and expel damaged molecules out of the cytoplasm in order to maintain homeostasis and to protect the cell against stress conditions (Takahashi et al., 2017). All together this suggests that NPC1 pathology is associated with increased autophagosome formation at the CPE cells in order to try to preserve homeostasis.

Next to autophagosomes, cells can also produce vesicles generated from endocytosis and endosomal compartments. Similar to autophagosomes, these vesicles may transport damaged molecules to the lysosomes or expel them out of the cell via exocytosis. Interestingly, we and others showed that the choroid plexus is able to release EVs, including exosomes, into the CSF (Grapp et al., 2013; Balusu et al., 2016b; Lepko et al., 2019). Moreover, we showed that inflammation has an effect on choroid plexus-derived EV release into the CSF and those EVs spread inflammation into the CNS. Here, we studied whether the increased inflammation and accumulation of autophagosomes in the CPE cells was reflected by changes in CSF EV amounts, size and activity. We show that larger and less CSF EVs are present in NPC1^–/–^ mice compared to NPC1^+/+^ mice, indicating that NPC1-loss and subsequent lipid dyshomeostasis affects dynamics of multiple vesicles that goes beyond LE/Lys which are known to accumulate free cholesterol and other lipids due to NPC1/2 dysfunction. The aberrant biogenesis of EVs observed in the CSF of NPC1^–/–^ mice is in line with the recent finding of a striking accumulation of multi-vesicular bodies in mouse NPC1^–/–^ microglia and the most significant change of CD63, a late endosomal and exosomal marker, among the NPC1^–/–^ microglia proteomic signature (Colombo et al., 2021). Next, analyzing the effect of NPC1^–/–^ versus NPC1^+/+^ choroid explant derived EVs revealed that NPC1^–/–^ EVs have pro-inflammatory and detrimental effects on brain cells both *in vitro* and *in vivo*. Indeed, NPC1^–/–^ choroid explant EVs induced increased gene expression of *Ccl3* and *Cxcl10* in MCCs cells. Moreover, LDH levels were hugely elevated in their supernatant compared to cells treated with wild type EVs which is indicative for cell damage. These results suggest that the choroid plexus of NPC1^–/–^ mice is able to produce EVs with pathological properties. This was further confirmed by our *in vivo* studies. Remarkably, the injection of EVs produced by NPC1^–/–^ choroid plexus into the striatum or PFC of wild type mice is enough to induce microgliosis and astrocytosis, two prominent pathological features of the NPC1 disease. Based on our TUNEL staining, no apoptotic cells were visible in the striatum or PFC of the injected mice. Meaning that a difference in apoptotic cell death cannot be the explanation for the observed difference in amount of microglia or astrocytes. Important to note is that we solely looked at the pathologic effect of purified EVs obtained from choroid plexus explants. Of course, also other components present in the CSF of NPC1^–/–^ mice may have pathologic effects on the brain.

In conclusion, our data indicate the NPC1 pathology is associated with changes at the choroid plexus, including enhanced inflammation, autophagosome accumulation and decreased EV secretion, and that these altered EVs might play a detrimental effect in the neuropathology of NPC1 disease by spreading neuroinflammation. These findings may open the way to new therapeutic strategies which may alter the production of the toxic CP-derived EVs.

## Materials and Methods

### Mice

Balb/cNctr-*Npc1^*m*1*N*^*/J mice aged 7–9 weeks were used. These mice harbor an insertion in chromosome 18, resulting in a premature truncation of the NPC1 protein deleting 11 out of 13 transmembrane domains. In all experiments age- and gender-matched wildtype littermates were used. All mice were housed with 14- to 10-h light and dark cycles and free access to food and water in SPF conditions. All experiments comply with the current laws of Belgium and were approved by the animal ethics committee of Ghent University.

### Cerebrospinal Fluid Isolation

Cerebrospinal fluid was obtained from the fourth ventricle via the cisterna magna puncture method as described previously (Liu and Duff, 2008; Brkic et al., 2015; Steeland et al., 2018). Briefly, borosilicate glass capillary tubes (Sutter Instruments; B100-75-15) were used to pull needles on the Sutter P-87 flaming micropipette puller (pressure 330 Pa, heat index 300). Mice were anesthetized with ketamine/xylazine and the incision site was sterilized with 70% ethanol. The cisterna magna was exposed by dissecting skin and muscle tissue on the posterior side of the skull. The head of the mouse was mounted at an angle of 135°, and CSF was collected from the fourth ventricle by puncturing the cisterna magna using the capillary needles.

### Tissue Sample Isolation

For RNA or protein analysis, mice were transcardially perfused with a D-PBS/heparin [0.2% heparin (5.000 IU/ml, Wockhardt)] supplemented with 0.5% bromophenol blue (Sigma). For choroid plexus explant experiments, mice were transcardially perfused with serum free DMEM-F12 medium (Gibco). Brain tissue was dissected out, and choroid plexus was obtained from fourth and lateral ventricles. For RNA or protein analysis the isolated choroid plexus was immediately snap-frozen in liquid nitrogen. For immunohistochemical analysis, mice were transcardially perfused with 4% PFA, brain samples were isolated, fixed in 4% PFA and processed for paraffin-embedding.

### RNA Isolation and Real-Time qPCR

RNA was isolated from choroid plexus using the Aurum total RNA Mini Kit (Bio-Rad), according to the manufacturer’s instructions. RNA concentration and purity were determined spectrophotometrically using the Nanodrop ND-1000 (Nanodrop Technologies, Thermo Scientific), and cDNA was synthesized with the SensiFAST^TM^ cDNA Synthesis Kit (Bioline). Real time quantitative polymerase chain reaction (RT-qPCR) was performed with the Light Cycler 480 system (Roche) using SensiFast SYBR No-Rox (Bio-Line). Expression levels were normalized to the expression of two or three most stable reference genes, determined using the geNorm Housekeeping Gene Selection Software (Vandesompele et al., 2002a, b). The sequences of the forward and reversed primers for the different genes are provided (Table 1).

**TABLE 1:** Overview of used RT-qPCR primer sequences.

Gene	Forward	Reverse
*Gapdh*	TGAAGCAGGCATCTGAGGG	CGAAGGTGGAAGAGTGGGAG
*Hprt*	AGTGTTGGATACAGGCCAGAC	CGTGATTCAAATCCCTGAAGT
*Rpl*	CCTGCTGCTCTCAAGGTT	TGGTTGTCACTGCCTCGTACTT
*Ubc*	AGGTCAAACAGGAAGACAGACGTA	TCACACCCAAGAACAAGCACA
*Ccl2*	TTAAAAACCTGGATCGGAACCAA	GCATTAGCTTCAGATTTACGGGT
*Ccl3*	TTCTCTGTACCATGACACTCTGC	CGTGGAATCTTCCGGCTGTAG
*Ccl5*	GCTGCTTTGCCTACCTCTCC	TCGAGTGACAAACACGACTGC
*Cldn5*	GCAAGGTGTATGAATCTGTGCT	GTCAAGGTAACAAAGAGTGCCA
*Cxcl10*	CCAAGTGCTGCCGTCATTTTC	GGCTCGCAGGGATGATTTCAA
*Cxcl11*	ACACTCCACGCTACCTTCTG	TGTGCCTCGTGATATTTGG
*Cdh1*	CAGGTCTCCTCATGGCTTTGC	CTTCCGAAAAGAAGGCTGTCC
*Il1*β	CACCTCACAAGCAGAGC ACAAG	GCATTAGAAACAGTCCA GCCCATAC
*Il6*	TAGTCCTTCCTACCCCAATTTCC	TTGGTCCTTAGCCACTCCTTC
*Ocln*	CCAGGCAGCGTGTTCCT	TTCTAAATAACAGTCACCT GAGGGC
*Tnf*	ACCCTGGTATGAGCCCATATAC	ACACCCATTCCCTTCACAGAG
*Zo1*	AGGACACCAAAGCATGTGAG	GGCATTCCTGCTGGTTACA

### Immunohistochemistry

For immunostaining on mouse brain sections, 5 μm sections were prepared from paraffin-embedded samples. Sections were dewaxed using Varistain (Thermo Fisher Scientific), followed by an antigen retrieval step using citrate buffer (Vector; H-3300) and wash steps with PBS-T (PBS + 0.5% Triton X-100) for the colorimetric stainings (IBA1 and GFAP) and PBS for the fluorescent stainings (ZO1, OCL, and CDH1). Next, endogenous peroxidase activity was blocked with 3% H_2_O_2_ in methanol for 10 min for the colorimetric stainings. Samples were blocked with bovine serum albumin (BSA) and 5% normal goat serum in PBT (0.5% BSA, 0.02% Triton X-100 in PBS) for 30 min at RT. For Zo-1 stainings 5% NGS in PBS and for OCLN and CDH1 stainings 5% NGS in PBS-T was used as blocking buffer. Next overnight incubation at 4°C with primary antibody against ZO1 (1:500; Invitrogen; 617300), OCL (1:100; Invitrogen; 33-1500), CDH1 (1:500; Becton Dickinson Benelux NV; 610181), GFAP (1:1000; Agilent; Z033429-2), or IBA1 (1:500; Wako Chemicals, 019-19741) was performed. After ON incubation, slides were washed with PBS and incubated with secondary antibody (1:100, Dylight633). For the IBA1 and GFAP staining, visualization was done using ABC (Vector; PK6100) and DAB. For fluorescent stainings, slides were stained with DAPI (1:1000) and mounted with n-propyl mounting medium. For colorimetric stainings, slides were stained with haematoxyline and mounted with Entellan. Image acquisition of fluorescently stained sections was performed using a LSM780 confocal microscope (Carl Zeiss Microscopy GmbH), IBA1 and GFAP slides were scanned using a slide scanner (Zeiss, Axio Scan) and analyzed with Zen software (Carl Zeiss Microscopy GmbH, 2012). Quantification of IBA positive and GFAP positive cells was performed using ImageJ software (version 1.53c, National Institutes of Health) and reported as number of cells per μm^2^).

### Analysis of Blood-Cerebrospinal Fluid Barrier Integrity

Fifteen minutes before CSF isolation, mice were intravenously (iv) injected with 250 mg/kg FITC-labeled dextran (4 kDa, Sigma). A CSF sample of 2 μl was diluted 50 times in PBS, and leakage from the blood into the CSF was determined by measuring the fluorescence in the diluted CSF samples (λ_*ex*_/λ_*em*_ = 485/520 nm) using the FLUOstar omega (Isogen LifeScience).

### Transmission Electron Microscopy

The choroid plexus of the fourth ventricle was isolated from 7 to 9 week old NPC1^–/–^ and NPC1^+/+^ mice. Primary fixation of the isolated choroid plexus was performed in a solution of 2.5% glutaraldehyde and 4% formaldehyde dissolved in 0.1 M sodium cacodylate buffer, pH7.2 for 4 h at RT followed by fixation O/N at 4°C. After washing, the samples were post-fixed in 1% OsO_4_ with 1.5% K_3_Fe (CN)_6_ in 0.1 M sodium cacodylate buffer at RT for 1 h. After washing in bidistilled H_2_O, samples were subsequently dehydrated through a graded ethanol series, including a bulk staining with 1% uranyl acetate at the 50% ethanol step followed by embedding in Spurr’s resin. Ultrathin sections of a gold interference color were cut using an ultramicrotome (Leica EM UC6), followed by a post-staining in a Leica EM AC20 for 40 min in uranyl acetate at 20°C and for 10 min in lead stain at 20°C. Sections were collected on formvar-coated copper slot grids. Grids were viewed with a JEM 1400plus transmission electron microscope (JEOL, Tokyo, Japan) operating at 80 kV.

### 3D Scanning Electron Microscopy

The choroid plexus of the fourth ventricle was dissected from NPC1^–/–^ mice of 9 weeks, and was immediately transferred into fixation buffer (4% PFA, EMS; 2.5% glutaraldehyde, EMS in 0.1 M Cacodylate buffer, pH 7.4). After overnight fixation at 4°C, samples were washed 3 × 5 min in cacodylate buffer and subsequently osmicated in 2% osmium (EMS), 1.5% ferrocyanide in cacodylate buffer for 1 h, and then extensively washed in ultrapure water (UPW). This was followed by incubation in 1% thiocarbohydrazide (20 min), washes in UPW, and a second osmication in 2% osmium in UPW (30 min). The samples were washed 5 × 3 min in UPW and placed in uranyl acetate replacement stain (EMS), 1:3 in water at 4°C overnight. The following day after washing in UPW, they were stained with Walton’s lead aspartate stain for 30 min at 60°C. For this, a 30 mM L-aspartic acid solution was used to freshly dissolve lead nitrate (20 mM, pH 5.5). The solution was filtered before use. After the final washes, the samples were dehydrated using a series of ice-cold solutions of increasing EtOH concentration (30, 50, 70, 90, 2 × 100%), followed by two dehydrations of 10 min in propylene oxide. Subsequent infiltration with resin (Spurr’s; EMS) was done by first incubating the samples overnight in 50% resin in propylene oxide, followed by at least two changes of fresh 100% resin. Next, samples were embedded in fresh resin and cured in the oven at 65°C for 72 h.

For SBF-SEM imaging, the resin-embedded samples were mounted on an aluminum specimen pin (Gatan) using conductive epoxy (Circuit Works). The specimens were trimmed in a pyramid shape using an ultramicrotome (Ultracut; Leica), and the block surface was trimmed until smooth and at least a small part of tissue was present at the block face. Next, samples were coated with 5 nm Pt in a Quorum Q 150T ES sputter coater (Quorum Technologies). The aluminum pins were placed in the Gatan 3View2 in a Zeiss Merlin SEM for imaging at 1.6 kV with a Gatan Digiscan II ESB detector. The Gatan 3View2 was set to 300 sections of 70 nm.

For FIB-SEM imaging, the pin was coated with 20 nm Pt in the sputter coater. FIB-SEM imaging was performed in a Zeiss Crossbeam 540 with Atlas 5 software. The Focused Ion Beam (FIB) was set to remove 5 nm sections by propelling Gallium ions at the surface. Imaging was done at 1.5 kV using an ESB (back-scattered electron) detector at a pixel size of 5 nm.

The resulting datasets were registered and converted to TIFF image stacks using IMOD^1^ and Fiji (Schindelin et al., 2012). Autophagosomes were segmented using Microscopy Image Browser (MIB)^2^ and visualization of labels and representation of the cell in 3D movies and snapshots was done in Imaris (BitPlane).

### Negative Staining of Extracellular Vesicles via Transmission Electron Microscopy

For the visualization of EVs in CSF, 5 μl of sample was spotted on a parafilm sheet. Formvar/C-coated hexagonal copper grids (EMS G200H-Cu), which were glow discharged for 10 s, were placed on top of the droplet for 1 min with the coated side of the grid down. The grids were washed five times in droplets of Milli-Q water, stained with 1% (w/v) uranyl acetate for 10 s and air dried for 24 h before imaging. Visualization of the samples was done using a JEM 1400plus transmission electron microscope (JEOL, Tokyo, Japan) operating at 80 kV.

### ExoView Analysis

We applied the ExoView technology using the ExoView Mouse Tetraspanin kit (EV-TETRA-M, NanoView Biosciences) and the ExoView R100 reader (NanoView Biosciences, Boston, MA, United States) to determine the amount of CD9 and CD81 positive EVs in CSF of NPC1^–/–^ and NPC1^+/+^ mice. In short, CSF samples are diluted to the desired concentration using incubation solution. The samples were incubated on the ExoView Tetraspanin Chip according to the manufacturer’s instructions and placed in a sealed 24-well plate for 16 h at RT. The chips were washed three times in incubation solution for 3 min each on an orbital shaker. Then, chips were incubated for 1 h with ExoView Tetraspanin Labeling Antibodies consisting of anti-CD81, and anti-CD9. The antibodies were diluted 1:600 in blocking solution. Afterward, the chips were washed three times with wash solution followed by a rinse in rinse solution and filtered water and dried. The chips were imaged with the ExoView R100 reader using the ExoScan acquisition software. Data were analyzed using ExoViewer (NanoView Biosciences).

### Choroid Plexus Explants

Choroid plexus explants were prepared as we described previously (Balusu et al., 2016b; Vandendriessche et al., 2021). In short, 9 weeks old NPC1^+/+^ and NPC1^–/–^ mice were anesthetized and transcardially perfused with serum free DMEM-F12 medium (11320074; Gibco) after which the choroid plexus (CP) tissue was dissected. The choroid plexus explants were incubated in 24-well plates containing 350 μl of Opti-MEM (11058-021; Gibco) medium [supplemented with penicillin/streptomycin (pen/strep; P4333; Sigma); non-essential amino-acids (NEAA; M-7145; Sigma), sodium pyruvate (S8636; Sigma), and L-glutamine (BE17-605F; Lonza)] per well for 16 h at 37°C and 5% CO2. Conditioned medium was collected and briefly centrifuged at 300 × *g* for 5 min and 4°C to eliminate cellular debris. A part of the supernatant was used to perform Bio-Plex cytokine analysis (Bio-Rad) according to the manufacturer’s instructions. The remaining supernatant (300 μl) was subjected to SEC purification followed by transfer of the EVs to the MCC.

### Extracellular Vesicle Purification From CP Explants Using Size Exclusion Chromatography

Conditioned medium of CP explant cultures (300 μl per explant) was collected after 16 h of incubation at 37°C and 5% CO2. Next, the conditioned medium was centrifuged at 300 × *g* for 5 min at 4°C to remove debris. The supernatant was diluted in PBS to obtain a final volume of 500 μl, after which the EVs were immediately (without intermediate storage) separated using the qEV original columns (qEV original/70 nm SEC columns; Izon Science) according to the manufacturer’s instructions. After discarding the void volume (3 ml), fractions 2 and 3 were collected, pooled and concentrated to approximately 30 μl using the Amicon Ultra-0.5 Centrifugal Filter Unit with Ultracel-10 membrane (UFC501024; Amicon). Next, Opti-MEM was added to a final volume of 300 μl that was transferred onto the MCCs.

### Mixed Cortical Cultures

Mixed cortical cultures were prepared as we described previously (Balusu et al., 2016b). In short, the cerebral cortex and hippocampus were obtained from 3 to 5 neonatal P1/P2 C57BL/6J mice from which the meninges were removed. Next, tissues were finely minced with a surgical scalpel in cold PBS and samples were centrifuged at 300 × *g* for 5 min after which they were digested with 0.25% trypsin (T-4424; Sigma) for 15 min in a water bath at 37°C. Cells were again centrifuged at 300 × *g* for 5 min and washed twice with ice-cold PBS. Cells were resuspended in DMEM (41965-062; Gibco) culture medium [supplemented with 10% fetal bovine serum (FBS); pen/strep (P4333; Sigma); NEAA (NEAA; M-7145; Sigma); sodium pyruvate (S8636; Sigma); and L-glutamine (BE17-605F; Lonza)] and plated directly in 24-well plates coated with 0.1% poly-L-lysine (P6282, Sigma). Cells were maintained in standard tissue culture conditions and 50% of the medium was replaced every 2 days. When the cells reached confluence, they were incubated for 24 h at 37°C and 5% CO2 with SEC-purified EVs separated from the supernatant of choroid plexus explants derived NPC1^+/+^ or NPC1^–/–^ mice of 9 weeks. Next, conditioned medium was collected and stored at −80°C until LDH measurement and cytokine/chemokine analysis were performed. TRIzol reagent (250 μl/well; 15596018; Gibco) was added to the cells whereafter they were stored at −20°C until RNA isolation and qPCR analysis.

### Lactate Dehydrogenase Measurement

A colorimetric assay was performed for the quantification of cell death and cell lysis, based on the measurement of LDH released from the cytosol of damaged cells into the supernatant. For this the cytotoxicity detection kit (Roche) was used according to the manufacturer’s instructions. In short, 250 μl catalyst diluted in dH_2_O was mixed per 11.25 ml of dye solution. Samples were centrifuged at 1100 rpm for 5 min to remove cell debris. Next, 100 μl of samples was mixed with 100 μl of reaction mixture and incubated for 30 min at RT. After the addition of 50 μl stop solution (1 M HCl), the absorbance was measured at 490 nm and 680 nm.

### Injections in Striatum and Prefrontal Cortex

For injections in the striatum and PFC, mice were anesthetized with isoflurane and mounted on a stereotactic frame. A constant body temperature of 37°C was maintained using a heating pad. Injection coordinates were measured relative to the bregma intersection and were determined using the Franklin and Paxinos mouse brain atlas. For striatum injection: anteroposterior 0.02 cm, mediolateral 0.2 cm, dorsoventral −0.26 cm. For PFC injection: anteroposterior 0.2 cm, mediolateral −0.025 cm, dorsoventral 0.23 cm. By using a Hamilton needle, 2 μl was injected at a rate of 0.4 μl/min and at a concentration of 2.28 × 10^10^ particles/ml in the case of the NPC1^+/+^ set-up and at a concentration of 1.9 × 10^10^ particles/ml in the NPC1^–/–^ set-up.

### Statistical Analysis

Statistical analyses were performed using GraphPad Prism 7.0 (GraphPad Software, Inc.). Bars represent mean ± SEM. qPCR data were analyzed with an unpaired *t*-test unless mentioned differently. For the analysis of the blood-CSF barrier permeability a one-way ANOVA was used and for the Bio-Plex assay a Mann–Whitney *U*-test was used. Significance levels are indicated ^∗^0.01 ≤ *p* < 0.05; ^∗∗^0.001 ≤ *p* < 0.01; ^∗∗∗^0.001 ≤ *p* < 0.0001; and ^****^p < 0.0001.

## Data Availability Statement

The original contributions presented in the study are included in the article/Supplementary Material, further inquiries can be directed to the corresponding author/s.

## Ethics Statement

The animal study was reviewed and approved by the Ethics Committee of Ghent University.

## Author Contributions

LV, CVC, SH and RV designed the experiments. LH, CV, KD, GV, EV, JC, CVC, JX, and WC performed the experiments. AK and PB performed the SBF-SEM and FIB-SEM. RD performed the EM. LV, and RV wrote the manuscript. All authors reviewed the manuscript before submission.

## Conflict of Interest

The authors declare that the research was conducted in the absence of any commercial or financial relationships that could be construed as a potential conflict of interest.

## Publisher’s Note

All claims expressed in this article are solely those of the authors and do not necessarily represent those of their affiliated organizations, or those of the publisher, the editors and the reviewers. Any product that may be evaluated in this article, or claim that may be made by its manufacturer, is not guaranteed or endorsed by the publisher.

## Footnotes

1
http://bio3d.colorado.edu/imod/

2
http://mib.helsinki.fi/

## Supplementary Material

Supplementary Figure 1 Increased expression of type I IFN response genes in the choroid plexus of NPC1^–/–^ mice compared to NPC1^+/+^ littermates. qRT-PCR analysis of choroid plexus isolated from NPC1^+/+^ (*n* = 6; black) and NPC1^–/–^ (*n* = 6; white) of 7 weeks old mice. Results are represented relative to the NPC1^+/+^ condition. Data are shown as mean ± SEM. Statistical analyses on datasets were performed by Mann–Whitney test. Asterisks indicate statistical significance (^∗^*p* < 0.05, ^∗∗^*p* < 0.01).

Supplementary Figure 2 Representative images of TUNEL staining (red) of the corpus striatum and prefrontal cortex of wild type mice injected with EVs isolated of choroid plexus explants of NPC1^+/+^ and NPC1^–/–^. Cell nuclei are counterstained with Hoechst (blue) (*n* = 4–5). Scale bar represents 200 μm.
