# Supplementary figures and images for: Involvement of the Choroid Plexus in the Pathogenesis of Niemann-Pick Disease Type C
Source: Front Cell Neurosci. 2021 Oct 15;15:757482. doi: 10.3389/fncel.2021.757482 (PMC8555471; doi:10.3389/fncel.2021.757482)

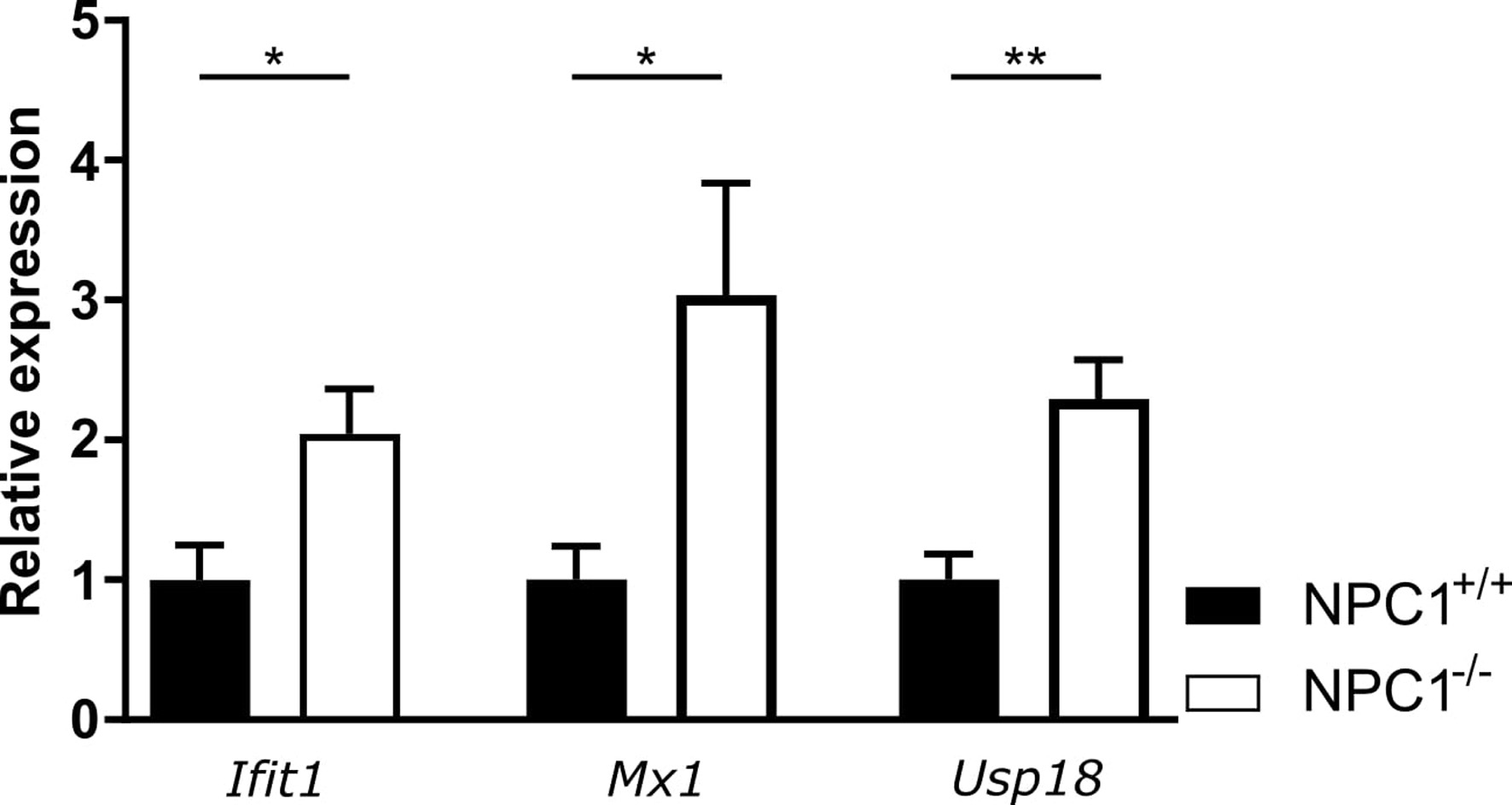

Supplement: Supplementary Figure 1 — Increased expression of type I IFN response genes in the choroid plexus of NPC1–/– mice compared to NPC1+/+ littermates. qRT-PCR analysis of choroid plexus isolated from NPC1+/+ (n = 6; black) and NPC1–/– (n = 6; white) of 7 weeks old mice. Results are represented relative to the NPC1+/+ condition. Data are shown as mean ± SEM. Statistical analyses on datasets were performed by Mann–Whitney test. Asterisks indicate statistical significance (∗p < 0.05, ∗∗p < 0.01). [file Image_1.JPEG]

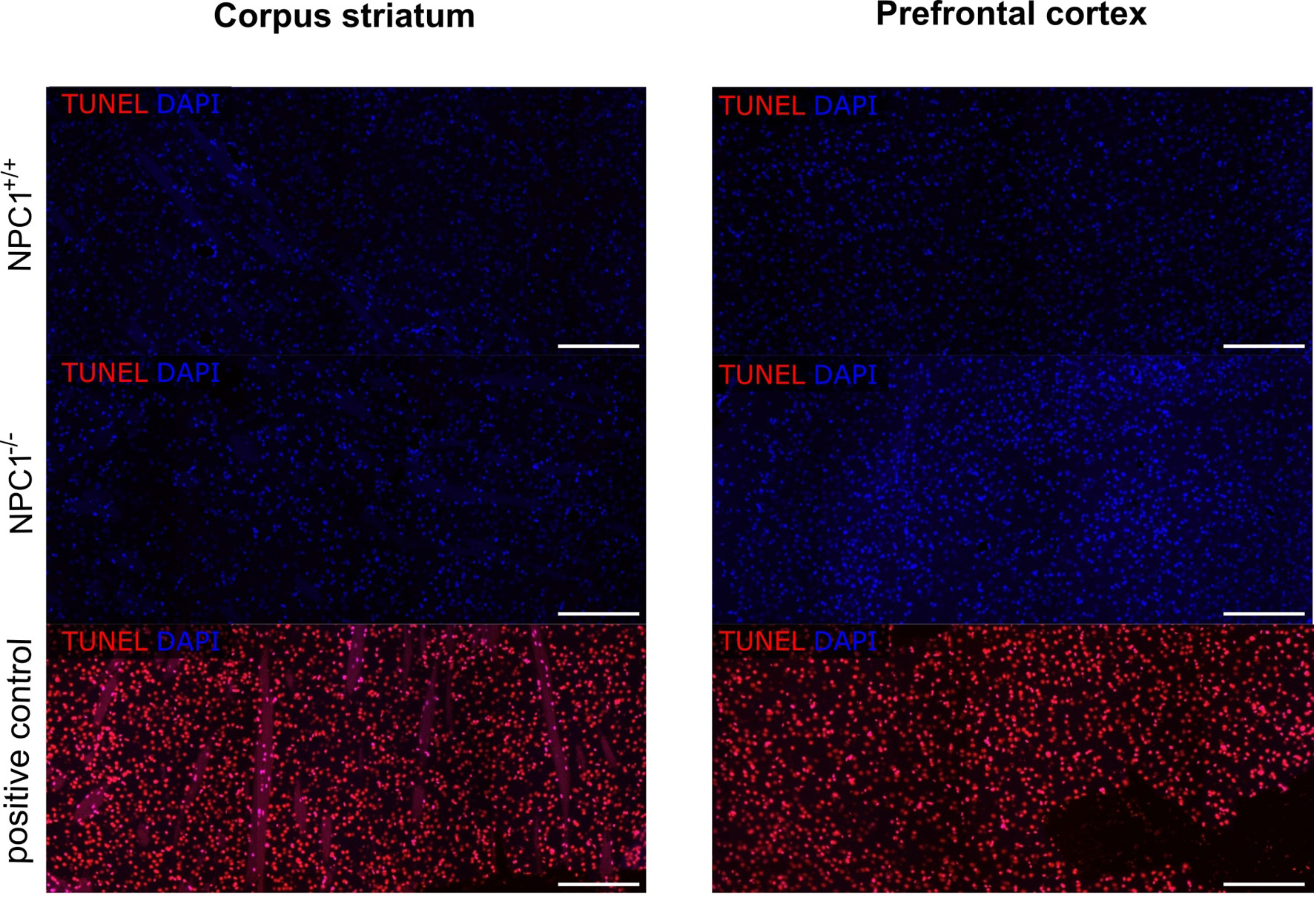

Supplement: Supplementary Figure 2 — Representative images of TUNEL staining (red) of the corpus striatum and prefrontal cortex of wild type mice injected with EVs isolated of choroid plexus explants of NPC1+/+ and NPC1–/–. Cell nuclei are counterstained with Hoechst (blue) (n = 4–5). Scale bar represents 200 μm. [file Image_2.JPEG]
